# Step-by-step state-selective tracking of fragmentation dynamics of water dications by momentum imaging

**DOI:** 10.1038/s41467-022-32836-6

**Published:** 2022-09-01

**Authors:** Travis Severt, Zachary L. Streeter, Wael Iskandar, Kirk A. Larsen, Averell Gatton, Daniel Trabert, Bethany Jochim, Brandon Griffin, Elio G. Champenois, Matthew M. Brister, Dylan Reedy, Demitri Call, Richard Strom, Allen L. Landers, Reinhard Dörner, Joshua B. Williams, Daniel S. Slaughter, Robert R. Lucchese, Thorsten Weber, C. William McCurdy, Itzik Ben-Itzhak

**Affiliations:** 1 J. R. Macdonald Laboratory, Physics Department, Kansas State University, Manhattan, KS 66506 USA; 2 Chemical Sciences Division, Lawrence Berkeley National Laboratory, Berkeley, CA 94720 USA; 3 Department of Chemistry, University of California, Davis, CA 95616 USA; 4 Graduate Group in Applied Science and Technology, University of California, Berkeley, CA 94720 USA; 5 Department of Physics, Auburn University, Auburn, AL 36849 USA; 6 Institut für Kernphysik, Goethe-Universität, Max-von-Laue-Straße 1, 60438 Frankfurt am Main, Germany; 7 Department of Physics, University of Nevada Reno, Reno, NV 89557 USA

**Keywords:** Atomic and molecular interactions with photons, Reaction kinetics and dynamics

## Abstract

The double photoionization of a molecule by one photon ejects two electrons and typically creates an unstable dication. Observing the subsequent fragmentation products in coincidence can reveal a surprisingly detailed picture of the dynamics. Determining the time evolution and quantum mechanical states involved leads to deeper understanding of molecular dynamics. Here in a combined experimental and theoretical study, we unambiguously separate the sequential breakup via D^+^ + OD^+^ intermediates, from other processes leading to the same D^+^ + D^+^ + O final products of double ionization of water by a single photon. Moreover, we experimentally identify, separate, and follow step by step, two pathways involving the b ^1^Σ^+^ and a ^1^Δ electronic states of the intermediate OD^+^ ion. Our classical trajectory calculations on the relevant potential energy surfaces reproduce well the measured data and, combined with the experiment, enable the determination of the internal energy and angular momentum distribution of the OD^+^ intermediate.

## Abstract (web summary)

Determining the time evolution of reactions at the quantum mechanical level improves our understanding of molecular dynamics. Here, authors separate the breakup of water, one bond at a time, from other processes leading to the same final products and experimentally identify, separate, and follow step by step two breakup paths of the transient OD^+^ fragment.

## Introduction

The measurement of reaction dynamics occurring on the femtosecond time scale has long been the target of various kinds of time-resolved spectroscopies and, more recently, has been accomplished with ultrafast X-ray measurements. Those measurements have exploited pump-probe strategies^1^, transient absorption^2^, and time-resolved X-ray scattering^3,4^, among other methods. Combining time-resolved X-ray measurements with momentum imaging coincidence detection^5–7^ that gives direct access to state-selective ionization and detailed insight into dissociation dynamics in the molecular frame remains a challenge. Recent interest in such time-resolved spectroscopies extends to the IR, UV and VUV regimes^8–12^.

Although it would seem intuitive that time resolution is necessary to see the steps in the unimolecular reactions that have frequently been the subjects of momentum imaging coincidence experiments, it was demonstrated that such experiments, without time-resolved X-ray or laser pulses, can in fact be used to distinguish the steps in a sequential reaction^13–21^. ﻿Recently, the native-frames analysis method was introduced^21,22^﻿, and the authors showed that photo-induced momentum imaging observations can resolve the sequence of events in the dissociation of a molecule involving vibrational and rotational dynamics following multiple ionization.

Here, the goal is to take a step further by combining such measurements with detailed ab initio theoretical calculations of the multiple Born–Oppenheimer potential surfaces governing such a reaction and the nuclear dynamics on those surfaces. With this methodology, the spectral signatures of the steps of a reaction, including nonadiabatic transitions that occur in those steps, can be identified experimentally. The expected result is a clear picture, not only of the steps, but of the electronic states of the transients involved in them.

In the present work, we accomplish the goal stated above. Specifically, we track sequential fragmentation step-by-step, by combining a kinematically complete measurement of all reaction products following single-photon absorption, computing the classical trajectories on the relevant potential-energy surfaces, and employing the native-frames analysis to separate the measured data into the steps of the process. Moreover, we are able to observe the internal-energy distribution of the intermediate molecule, which has sufficient energy to predissociate—demonstrating the power of the presented methodology.

## Results

### Sequential fragmentation of water

To explore the molecular dynamics causing sequential fragmentation we study, as a test case, the heavy water molecule that fragments into D^+^ + D^+^ + O(^3^P) + 2*e*^−^ following the absorption of a single photon. We focus on sequential fragmentation via an OD^+^ intermediate, which follows the steps listed below

1 $${{{{{{{{\rm{D}}}}}}}}}_{2}{{{{{{{{\rm{O}}}}}}}}}^{2+}\to {{{{{{{{\rm{D}}}}}}}}}_{{{{{{{{\rm{I}}}}}}}}}^{+}+{{{{{{{{\rm{OD}}}}}}}}}_{{{{{{{{\rm{II}}}}}}}}}^{+}\quad ({{{{{{{\rm{Step}}}}}}}}\,1)$$

2 $$\quad {{{{{{{{\rm{OD}}}}}}}}}_{{{{{{{{\rm{II}}}}}}}}}^{+}\to {{{{{{{{\rm{D}}}}}}}}}_{{{{{{{{\rm{II}}}}}}}}}^{+}+{{{{{{{\rm{O}}}}}}}}{\left(\right.}^{3}\left.{{{{{{{\rm{P}}}}}}}}\right)\quad ({{{{{{{\rm{Step}}}}}}}}\,2)\,.$$

Note that the D^+^ fragments are labeled I and II according to their ejection order in the sequential process, i.e., labeled by their fragmentation step according to Eq. (1) and Eq. (2), respectively (a notation used throughout). Despite the fact that these D atoms are indistinguishable, we show below that the sequential breakup step, i.e., the ejection order, can be associated with each detected D^+^ fragment.

It was suggested by Streeter et al.^23^, who identified many measured concerted fragmentation paths in H_2_O^24^, that one of the reaction pathways leading to the H^+^ + H^+^ +  O(^3^P) final state involves sequential breakup via an OH^+^ intermediate. Specifically, they speculated that a feature in the data, having broad angular spread between the protons’ ejection directions, may be due to transitions involving the 2 ^1^A_1_ state of the water dication, which undergoes two-body breakup to H^+^+ OH^+^(b ^1^Σ^+^). This b ^1^Σ^+^ state of OH^+^ is known to predissociate into H^+^ + O(^3^P) via a crossing with the A ^3^Π state^25,26^. We validate the sequential-breakup path described above and identify an unexpected additional sequential fragmentation pathway of D_2_O^2+^. Moreover, we reveal details on each step of these stepwise reaction dynamics, and also measure the excess internal energy above the dissociation limit of the metastable intermediate OH^+^ molecule.

### First fragmentation step

**Fig. 1:**
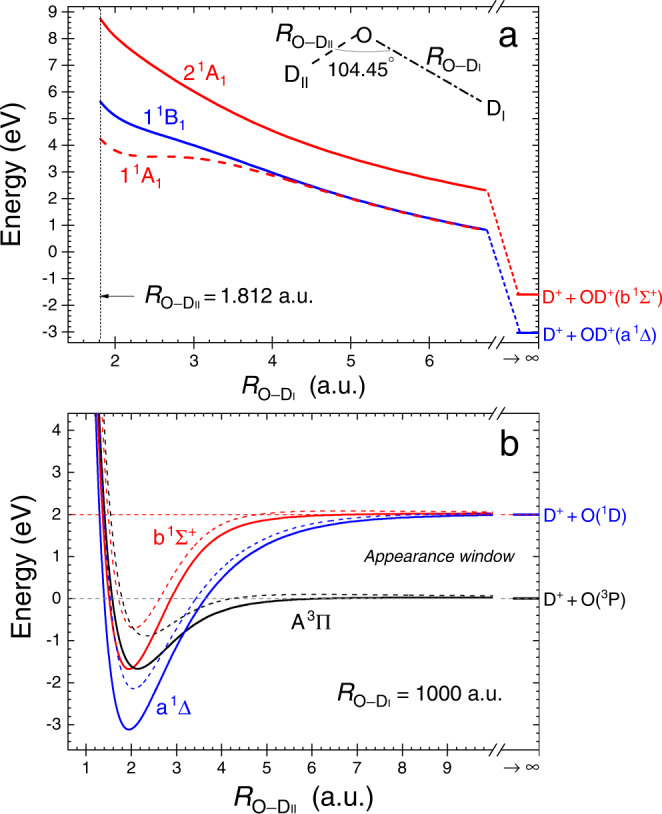
Potential energy surfaces of water dication, $V({R}_{{{{{{{{\rm{O}}}}}}}}-{{{{{{{{\rm{D}}}}}}}}}_{{{{{{{{\rm{I}}}}}}}}}},{R}_{{{{{{{{\rm{O}}}}}}}}-{{{{{{{{\rm{D}}}}}}}}}_{{{{{{{{\rm{II}}}}}}}}}})$, at the equilibrium angle. Cuts through the potential energy surfaces, relevant for sequential fragmentation of D_2_O^2+^ via OD${}_{{{{{{{{\rm{II}}}}}}}}}^{+}$ intermediate, showing **a** the asymmetric stretch of D_2_O^2+^ for a fixed bond angle (104.45^∘^) and internuclear distance (${R}_{{{{{{{{\rm{O}}}}}}}}-{{{{{{{{\rm{D}}}}}}}}}_{{{{{{{{\rm{II}}}}}}}}}}$ = 1.812 a.u.) of the other O–D bond for the three states of D_2_O^2+^ that produce the diatomic intermediate which further dissociates (see text), and **b** the OD${}_{{{{{{{{\rm{II}}}}}}}}}^{+}$ potentials when the interaction with the other D${}_{{{{{{{{\rm{I}}}}}}}}}^{+}$ is negligible (${R}_{{{{{{{{\rm{O}}}}}}}}-{{{{{{{{\rm{D}}}}}}}}}_{{{{{{{{\rm{I}}}}}}}}}}$ = 1000 a.u.), where solid lines are for *J* = 0, while the effective radial potentials for *J* = 30 are shown as dashed lines. The states in (a) plotted as solid blue and solid red curves produce the diatomic ion with enough internal energy to predissociate via the A ^3^Π state. The energy scale is relative to the D^+^ + D^+^ + O(^3^P) dissociation limit. Note that on this energy scale the asymmetric stretch limits for the 1 ^1^B _1_ and 2 ^1^A_1_ states are −3.021 and −1.619 eV, respectively. Source data are provided as a Source Data file.

The first step in sequential breakup requires one O–D_I_ bond to break, while the OD${}_{{{{{{{{\rm{II}}}}}}}}}^{+}$ fragment remains bound. In Fig. 1a, we show a cut of the relevant potential energy surfaces (PES) of D_2_O^2+^ leading to D${}_{{{{{{{{\rm{I}}}}}}}}}^{+}$ + OD${}_{{{{{{{{\rm{II}}}}}}}}}^{+}$ breakup along the dissociation of one D^+^ at the equilibrium bond angle of neutral water, which is the relevant angle in the Franck-Condon approximation. These states of D_2_O^2+^ correlate with the b ^1^Σ^+^ and a ^1^Δ states of OD^+^, which are predissociative via a crossing with the A ^3^Π state of OD^+^.

Figure 1b displays cuts in a few PES of D_2_O^2+^ for the same bond angle, but when one D${}_{{{{{{{{\rm{I}}}}}}}}}^{+}$ is at 1000 a.u. and therefore its interaction with the remaining OD${}_{{{{{{{{\rm{II}}}}}}}}}^{+}$ is negligible. These cuts are effective radial potentials for fixed *J* of the OD${}_{{{{{{{{\rm{II}}}}}}}}}^{+}$ states correlating with the D${}_{{{{{{{{\rm{II}}}}}}}}}^{+}$ + O(^3^P) and D${}_{{{{{{{{\rm{II}}}}}}}}}^{+}$ + O(^1^D) dissociation limits, which are involved in the predissociation of the intermediate OD${}_{{{{{{{{\rm{II}}}}}}}}}^{+}$ molecule.

### Second fragmentation step

The essence of the second step of the sequential fragmentation revealed in our experiment is the dissociation of the OD${}_{{{{{{{{\rm{II}}}}}}}}}^{+}$ ion produced in the first step in its b ^1^Σ^+^ and a ^1^Δ states by a spin-orbit induced transition to the A ^3^Π state. Only if the OD${}_{{{{{{{{\rm{II}}}}}}}}}^{+}$ ion is produced in rovibrationally excited states above the effective radial barrier to the dissociation limit of the A ^3^Π state, shown in Fig. 1b, can it be predissociated by this transition, and these dynamics leave clear signatures in the momenta of the final products.

Using the PES information detailed above, we model the reaction dynamics by the propagation of ensembles of classical trajectories on the relevant potential surface for a few picoseconds, at which time the OD${}_{{{{{{{{\rm{II}}}}}}}}}^{+}$ population is evaluated. We assume that all OD${}_{{{{{{{{\rm{II}}}}}}}}}^{+}$ ions with internal energy above the D${}_{{{{{{{{\rm{II}}}}}}}}}^{+}$ + O(^3^P) dissociation limit and zero rotational energy, i.e., between the two horizontal dashed lines in Fig. 1b labeled appearance window, predissociate to D${}_{{{{{{{{\rm{II}}}}}}}}}^{+}$ + O fragments within a few picoseconds due to spin-orbit coupling with the A ^3^Π state^25,26^. Nonzero rotational angular momentum adds a centrifugal barrier to this picture for all three states of OD${}_{{{{{{{{\rm{II}}}}}}}}}^{+}$ in Fig. 1b, modifying the appearance window, and leaving a clear signature of rotational excitation in the kinetic energy of the atomic fragments produced by this mechanism, as we discuss below.

### Fragmentation experiment and analysis

To explore the sequential-breakup mechanism experimentally, we initiate fragmentation by the absorption of a 61 eV photon, thereby producing a doubly-ionized water molecule at a well-defined energy (24.1 eV) above the lowest dissociation limit, D^+^ + D^+^ + O(^3^P). We measure all four charged fragments in coincidence and determine their final momenta using a cold target recoil ion momentum spectroscopy (COLTRIMS) technique^5–7^. Specifically, we focus on events producing two electrons, two deuterons, and a neutral oxygen whose final momentum is evaluated using momentum conservation.

**Fig. 2:**
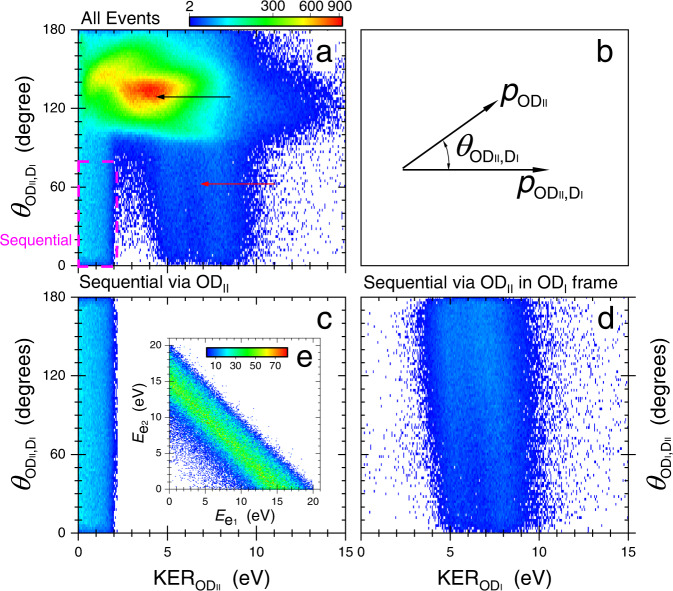
Native frames analysis of D^+^ + D^+^ + O breakup. **a** All the measured D^+^ + D^+^ + O events (shown using a $\sqrt{{{{{{{{\rm{counts}}}}}}}}}$ scale) as a function of the kinetic energy released upon OD${}_{{{{{{{{\rm{II}}}}}}}}}^{+}$ dissociation, KER${}_{{{{{{{{{\rm{OD}}}}}}}}}_{{{{{{{{\rm{II}}}}}}}}}}$, and the angle between the conjugate momenta, ${\theta }_{{{{{{{{{\rm{OD}}}}}}}}}_{{{{{{{{\rm{II}}}}}}}}},{{{{{{{{\rm{D}}}}}}}}}_{{{{{{{{\rm{I}}}}}}}}}}$. Sequential fragmentation events via D${}_{{{{{{{{\rm{I}}}}}}}}}^{+}$ + OD${}_{{{{{{{{\rm{II}}}}}}}}}^{+}$ are identified (see text) by the uniform angular distribution within the magenta-dashed rectangle (i.e., KER${}_{{{{{{{{{\rm{OD}}}}}}}}}_{{{{{{{{\rm{II}}}}}}}}}}\, < \, 2.3$ eV and 0${}^{\circ }\, < \, {\theta }_{{{{{{{{{\rm{OD}}}}}}}}}_{{{{{{{{\rm{II}}}}}}}}},{{{{{{{{\rm{D}}}}}}}}}_{{{{{{{{\rm{I}}}}}}}}}}\, < \, 8{0}^{\circ }$). **b** Schematic diagram of the conjugate momenta and the angle between them (see Eqs. (5) and (6) in the “Methods” section). Sequential breakup via OD${}_{{{{{{{{\rm{II}}}}}}}}}^{+}$ analyzed in the **c** OD${}_{{{{{{{{\rm{II}}}}}}}}}^{+}$ and **d** OD${}_{{{{{{{{\rm{I}}}}}}}}}^{+}$ frames, i.e., the correct and wrong fragmentation-step order of the D^+^ fragments, respectively (see text). As detailed in the “Methods” section, analysis of the events in the wrong reference frame, as is the case shown in panel (**d**) and indicated by the different axis labels (KER${}_{{{{{{{{{\rm{OD}}}}}}}}}_{{{{{{{{\rm{I}}}}}}}}}}$ and ${\theta }_{{{{{{{{{\rm{OD}}}}}}}}}_{{{{{{{{\rm{I}}}}}}}}},{{{{{{{{\rm{D}}}}}}}}}_{{{{{{{{\rm{II}}}}}}}}}}$), yields a distribution significantly different than the one expected for sequential fragmentation^21,22^, specifically, a non-uniform angular distribution, a KER${}_{{{{{{{{{\rm{OD}}}}}}}}}_{{{{{{{{\rm{II}}}}}}}}}}$ that depends on the angle ${\theta }_{{{{{{{{{\rm{OD}}}}}}}}}_{{{{{{{{\rm{II}}}}}}}}},{{{{{{{{\rm{D}}}}}}}}}_{{{{{{{{\rm{I}}}}}}}}}}$, and a much higher than expected KER${}_{{{{{{{{{\rm{OD}}}}}}}}}_{{{{{{{{\rm{II}}}}}}}}}}$. This sequential fragmentation distribution, analyzed in the wrong D^+^ ejection order, is marked by a red arrow in panel (**a**), while concerted breakup is indicated by a black arrow. **e** Energy-correlation map of the ionized electrons associated with sequential fragmentation via D${}_{{{{{{{{\rm{I}}}}}}}}}^{+}$ + OD${}_{{{{{{{{\rm{II}}}}}}}}}^{+}$ (shown as counts). Source data are provided as a Source Data file.

We analyze the three-body fragmentation assuming an OD${}_{{{{{{{{\rm{II}}}}}}}}}^{+}$ intermediate using the native frames method^21,22^. In our present case, shown schematically in Fig. 2b, the conjugate momenta (of the Jacobi coordinates) associated with the first and second breakup steps are ${{{{{{{{\rm{p}}}}}}}}}_{{{{{{{{{\rm{OD}}}}}}}}}_{{{{{{{{\rm{II}}}}}}}}},{{{{{{{{\rm{D}}}}}}}}}_{{{{{{{{\rm{I}}}}}}}}}}$ and ${{{{{{{{\rm{p}}}}}}}}}_{{{{{{{{{\rm{OD}}}}}}}}}_{{{{{{{{\rm{II}}}}}}}}}}$, respectively, while ${\theta }_{{{{{{{{{\rm{OD}}}}}}}}}_{{{{{{{{\rm{II}}}}}}}}},{{{{{{{{\rm{D}}}}}}}}}_{{{{{{{{\rm{I}}}}}}}}}}$ is the angle between them—all defined in the “Methods” section.

### Identifying sequential fragmentation of water

Rotation of the intermediate diatomic ion in the fragmentation plane provides a clear signature of sequential fragmentation^15–18,21,22,27^. In the native frames analysis this rotation manifests itself as a nearly uniform $N({\theta }_{{{{{{{{{\rm{OD}}}}}}}}}_{{{{{{{{\rm{II}}}}}}}}},{{{{{{{{\rm{D}}}}}}}}}_{{{{{{{{\rm{I}}}}}}}}}})$ angular distribution if the predissociation lifetime is long enough so the rotation of OD${}_{{{{{{{{\rm{II}}}}}}}}}^{+}$ wipes out any initial angular preference. As detailed in the “Methods” section, in Fig. 2a we show the distinct uniform angular distribution of sequential fragmentation via D${}_{{{{{{{{\rm{I}}}}}}}}}^{+}$ + OD${}_{{{{{{{{\rm{II}}}}}}}}}^{+}$ (marked by the dashed-magenta rectangular boundary), which occurs at the expected kinetic energy release (KER) of OD${}_{{{{{{{{\rm{II}}}}}}}}}^{+}$ dissociation^25^, indicated by the appearance window in Fig. 1b.

Three observations are appropriate at this point: First, in Fig. 2c we show the complete angular distribution of this sequential-fragmentation channel of D_2_O^2+^, which was reconstructed taking advantage of the fact that $N({\theta }_{{{{{{{{{\rm{OD}}}}}}}}}_{{{{{{{{\rm{II}}}}}}}}},{{{{{{{{\rm{D}}}}}}}}}_{{{{{{{{\rm{I}}}}}}}}}})$ is nearly uniform^21,22^.

Second, one can associate each detected D^+^ with the relevant fragmentation step using the native frames analysis, as described in the “Methods” section. After correctly assigning which D^+^ comes from the OD^+^ intermediate, we combine the data for all plots presented below, i.e., we use all sequential events via OD${}_{{{{{{{{\rm{II}}}}}}}}}^{+}$.

**Fig. 3:**
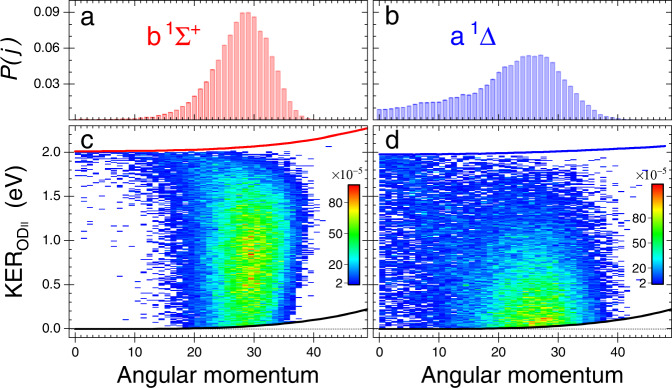
Normalized angular momentum distributions. These *P*(*J*) distributions (Σ_*j*_ *P*(*j*) = 1) are computed for the **a** b ^1^Σ^+^ and **b** a ^1^Δ states of OD^+^ populated during D_2_O^2+^ fragmentation (see text). Calculated angular momentum—KER${}_{{{{{{{{{\rm{OD}}}}}}}}}_{{{{{{{{\rm{II}}}}}}}}}}$ correlation maps for the **c** b ^1^Σ^+^ and **d** a ^1^Δ states. The lines in panels **c** and **d** represent the modification of the appearance window in Fig. 1b for nonzero rotational angular momentum by the addition of a centrifugal potential to both the singlet and triplet potential curves (specifically, A ^3^Π - black, b ^1^Σ^+^ - red, and a ^1^Δ - blue). In the absence of tunneling no dissociating trajectories can appear outside these lines. Source data are provided as a Source Data file.

Finally, we note that the high degree of rotational excitation in the intermediate OD${}_{{{{{{{{\rm{II}}}}}}}}}^{+}$ b ^1^Σ^+^ and a^1^Δ states found in the classical trajectory simulation, which leads to the signature of sequential breakup, has its origin in the strong force towards bond opening on the corresponding 2^1^A_1_ and ^1^B_1_ potential surfaces of D_2_O^2+^ upon the loss of two electrons. In the simplest picture of the electronic structure of water, these states are created by the loss of two lone-pair electrons. From simple molecular orbital considerations, losing one or two electrons out of the in-plane 3*a*_1_ lone-pair orbital should lead to an opening of the bond. Streeter et al.^23^ found that this torque is strong enough to cause the two D^+^ fragments, ejected initially near the 104° equilibrium bond angle in a concerted three-body fragmentation on these surfaces, to invert this angle to greater than 180^∘^ while they are still close to the oxygen atom. Similarly, trajectories leading to the two-body breakup into D${}_{{{{{{{{\rm{I}}}}}}}}}^{+}$ + OD${}_{{{{{{{{\rm{II}}}}}}}}}^{+}$ impart a strong torque on the OD${}_{{{{{{{{\rm{II}}}}}}}}}^{+}$ fragment, resulting in high rotational excitation. Gervais et al.^26^ also found high rotational excitation in diatomic ion fragments from HOD having insufficient internal energy to predissociate, with the highest being from these two dication states. For the trajectories that produce the OD${}_{{{{{{{{\rm{II}}}}}}}}}^{+}$ fragment with enough internal energy to predissociate via the A ^3^Π state, in our classical trajectory calculations these dynamics lead to angular momentum distributions peaking near *J* = 30 as shown in Fig. 3, i.e., even higher levels of rotational excitation than in ref. 26. We note that the angular distribution of the a^1^Δ state extends down to *J* = 0, while the b ^1^Σ^+^ state of OD${}_{{{{{{{{\rm{II}}}}}}}}}^{+}$ cannot be produced rotationally cold. Moreover, this rotational distribution leaves its signature in the kinetic energies of the atomic fragments after dissociation as we discuss further below.

### Dissociation limit of sequential fragmentation of water

**Fig. 4:**
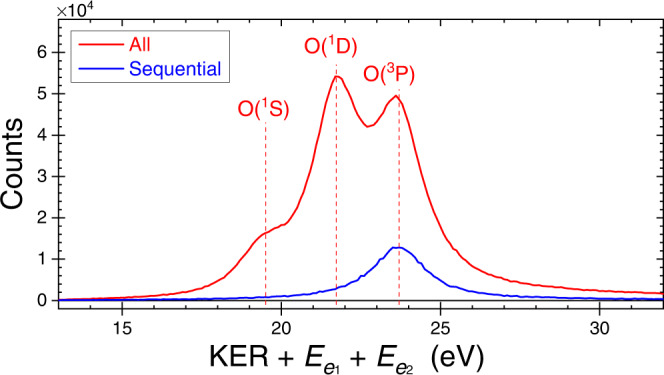
Total energy release in double photoionization of water. The measured distribution, *N*(KER + ${E}_{{e}_{1}}$ + ${E}_{{e}_{2}}$). The vertical lines indicate the expected energy of each D^+^ + D^+^ + O dissociation limit, which are labeled by the final state of the oxygen atom and shifted down by 0.4 eV (see text). Note that sequential fragmentation via OD${}_{{{{{{{{\rm{II}}}}}}}}}^{+}$ correlates only with the D^+^ + D^+^ + O(^3^P) dissociation limit. The broad energy distribution, clearly visible in the sequential breakup, is mainly due to the extended target along the light-propagation direction, energy resolution, and the spread in center-of-mass momentum that cannot be corrected for because the neutral oxygen fragment is not detected but rather evaluated from momentum conservation. Source data are provided as a Source Data file.

First, we confirm that the selected sequential fragmentation events lead to the expected D^+^ + D^+^+ O(^3^P) dissociation limit. To that end, in Fig. 4 we present all the measured D^+^ + D^+^+ O + 2*e*^−^ events (red-solid line), resulting from the absorption of a single 61 eV photon, as a function of the total energy release, i.e., *E*_release_ = KER + ${E}_{{e}_{1}}$ + ${E}_{{e}_{2}}$, where ${E}_{{e}_{1}}$ or ${E}_{{e}_{2}}$ is the kinetic energy of an electron in the continuum. Essentially, the measured *E*_release_ provides information about the dissociation limit, thus allowing one to determine the internal energy of the oxygen fragment. We evaluated the expected value of *E*_release_ for each dissociation limit from the known photon energy, the complete dissociation energy of water, and the initial state (i.e., ground state) of the heavy water molecule—those locations are marked by vertical lines and labeled by the oxygen final state in Fig. 4—though with a small shift down, ∼0.4 eV, due to experimental uncertainties (see ‘Experimental method’ in the “Methods” section for details). It is evident from the figure that the likelihood of fragmentation to the lowest two dissociation limits, i.e., O(^3^P) and O(^1^D), is approximately equal, and each of them is more likely than a breakup to the O(^1^*S*) limit^24^. In the same figure we also plot (blue line) the sequential events via OD^+^, selected by KER${}_{{{{{{{{{\rm{OD}}}}}}}}}_{{{{{{{{\rm{II}}}}}}}}}}\, < \, 2.3$ eV (see ‘Native frames analysis’ in the “Methods” section). Figure 4 clearly shows that the sequential fragmentation via OD${}_{{{{{{{{\rm{II}}}}}}}}}^{+}$ leads solely to the D^+^ + D^+^ + O(^3^P) dissociation limit. We also note that the electrons associated with this sequential breakup exhibit an energy sharing that is typical for direct double ionization involving no auto-ionization, as shown in Fig. 2e.

### Sequential fragmentation pathways

As mentioned briefly in the introduction, Streeter et al.^23^ suggested that the OD${}_{{{{{{{{\rm{II}}}}}}}}}^{+}$(b ^1^Σ^+^) is the intermediate state that predissociates, due to spin-orbit coupling with the A ^3^Π state (shown in Fig. 1b)^25,26^, into D${}_{{{{{{{{\rm{II}}}}}}}}}^{+}$ + O(^3^P) a few picoseconds after the D_2_O^2+^ breaks into D${}_{{{{{{{{\rm{I}}}}}}}}}^{+}$ + OD${}_{{{{{{{{\rm{II}}}}}}}}}^{+}$(b ^1^Σ^+^). Inspection of Fig. 1a indicates that the b ^1^Σ^+^ state of OD${}_{{{{{{{{\rm{II}}}}}}}}}^{+}$ correlates with the 2 ^1^A_1_ state of D_2_O^2+^. If the latter state is populated by a vertical transition from the D_2_O ground state (i.e., removing two electrons without changing the internuclear distances or bond angle, which is a reasonable approximation for ionization by a single photon), then a KER of about 7 eV is expected in the first fragmentation step, D${}_{{{{{{{{\rm{I}}}}}}}}}^{+}$ + OD${}_{{{{{{{{\rm{II}}}}}}}}}^{+}$(b ^1^Σ^+^), en route to D^+^ + D^+^ + O(^3^P).

**Fig. 5:**
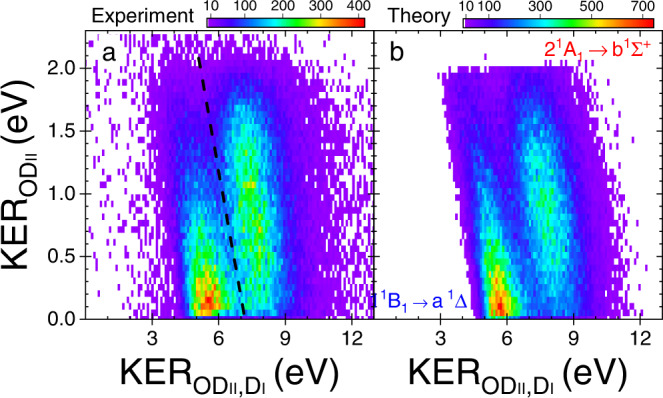
The KER correlation maps of sequential fragmentation of water dications. The measured yield of D_2_O^2+^ breakup into D^+^ + D^+^ + O(^3^P) as a function of KER${}_{{{{{{{{{\rm{OD}}}}}}}}}_{{{{{{{{\rm{II}}}}}}}}},{{{{{{{{\rm{D}}}}}}}}}_{{{{{{{{\rm{I}}}}}}}}}}$ and KER${}_{{{{{{{{{\rm{OD}}}}}}}}}_{{{{{{{{\rm{II}}}}}}}}}}$: **a** Experiment and **b** Theory. The two panels nicely match each other, aided by the choice of similar statistics in the theory to match the experimental data quality. The black-dashed line in panel (**a**) is used to separate the two sequential fragmentation paths (see text), specifically 2 ^1^A_1_ →  b ^1^Σ^+^ (right) from 1 ^1^B_1_ →  a ^1^Δ (left). This visualization of our pathway separation method, though correct, is simplified in practice by plotting the same data as a function of KER${}_{{{{{{{{{\rm{OD}}}}}}}}}_{{{{{{{{\rm{II}}}}}}}}},{{{{{{{{\rm{D}}}}}}}}}_{{{{{{{{\rm{I}}}}}}}}}}$ + KER${}_{{{{{{{{{\rm{OD}}}}}}}}}_{{{{{{{{\rm{II}}}}}}}}}}$ and KER${}_{{{{{{{{{\rm{OD}}}}}}}}}_{{{{{{{{\rm{II}}}}}}}}},{{{{{{{{\rm{D}}}}}}}}}_{{{{{{{{\rm{I}}}}}}}}}}$ − KER${}_{{{{{{{{{\rm{OD}}}}}}}}}_{{{{{{{{\rm{II}}}}}}}}}}$, i.e., a 45^∘^ rotation of the spectrum in panel (**a**). Then, we project it onto the KER = KER${}_{{{{{{{{{\rm{OD}}}}}}}}}_{{{{{{{{\rm{II}}}}}}}}},{{{{{{{{\rm{D}}}}}}}}}_{{{{{{{{\rm{I}}}}}}}}}}$ + KER${}_{{{{{{{{{\rm{OD}}}}}}}}}_{{{{{{{{\rm{II}}}}}}}}}}$ axis and associate events larger (smaller) than KER = 7.18 eV with the b ^1^Σ^+^ (or a ^1^Δ) channel (further details and the KER distributions are provided in Supplementary Note 1). Source data are provided as a Source Data file.

To verify this suggested pathway we plot in Fig. 5a the measured sequential fragmentation events selected by KER${}_{{{{{{{{{\rm{OD}}}}}}}}}_{{{{{{{{\rm{II}}}}}}}}}}\, < \,2.3$ eV, (see ‘Native frames analysis’ in the “Methods” section for details) leading to D^+^ + D^+^ + O(^3^P) as a function of KER${}_{{{{{{{{{\rm{OD}}}}}}}}}_{{{{{{{{\rm{II}}}}}}}}},{{{{{{{{\rm{D}}}}}}}}}_{{{{{{{{\rm{I}}}}}}}}}}$ and KER${}_{{{{{{{{{\rm{OD}}}}}}}}}_{{{{{{{{\rm{II}}}}}}}}}}$, i.e., the KER in the first and second fragmentation steps, respectively. One can clearly see the feature centered about the expected KER${}_{{{{{{{{{\rm{OD}}}}}}}}}_{{{{{{{{\rm{II}}}}}}}}},{{{{{{{{\rm{D}}}}}}}}}_{{{{{{{{\rm{I}}}}}}}}}}$ with a broad, relatively flat, KER${}_{{{{{{{{{\rm{OD}}}}}}}}}_{{{{{{{{\rm{II}}}}}}}}}}$ distribution extending to about 2.1 eV. This clearly affirms the predicted sequential fragmentation path D_2_O^2+^(2 ^1^A_1_) → D${}_{{{{{{{{\rm{I}}}}}}}}}^{+}$ + OD${}_{{{{{{{{\rm{II}}}}}}}}}^{+}$(b ^1^Σ^+^) followed by OD${}_{{{{{{{{\rm{II}}}}}}}}}^{+}$(b ^1^Σ^+^) → D${}_{{{{{{{{\rm{II}}}}}}}}}^{+}$ + O(^3^P).

Note that there is a prominent second feature in Fig. 5a centered on a lower KER${}_{{{{{{{{{\rm{OD}}}}}}}}}_{{{{{{{{\rm{II}}}}}}}}},{{{{{{{{\rm{D}}}}}}}}}_{{{{{{{{\rm{I}}}}}}}}}}$ of about 5.5 eV. This is unexpected, considering the suggestion of Streeter et al.^23^ that a single electronic state is involved in sequential fragmentation. Moreover, the KER${}_{{{{{{{{{\rm{OD}}}}}}}}}_{{{{{{{{\rm{II}}}}}}}}}}$ distribution of this feature peaks near zero and tails off rapidly. These distinct features suggest different fragmentation pathways.

Examination of the potential energy along the asymmetric stretch coordinate, shown in Fig. 1a, indicates that the 1 ^1^B_1_ state of the dication, at the equilibrium ${R}_{{{{{{{{{\rm{OD}}}}}}}}}_{{{{{{{{\rm{I}}}}}}}}}}$ of water (1.812 a.u.), is about 5.5 eV above the D^+^ + D^+^ + O(^3^P) dissociation limit associated with the first fragmentation step, D${}_{{{{{{{{\rm{I}}}}}}}}}^{+}$ + OD${}_{{{{{{{{\rm{II}}}}}}}}}^{+}$(a ^1^Δ). Therefore, we attribute this lower KER${}_{{{{{{{{{\rm{OD}}}}}}}}}_{{{{{{{{\rm{II}}}}}}}}},{{{{{{{{\rm{D}}}}}}}}}_{{{{{{{{\rm{I}}}}}}}}}}$ feature to sequential fragmentation initiated by double ionization to the 1 ^1^B_1_ state, which is consistent with the results of our classical trajectories on the 1 ^1^B_1_ potential surface. This state, then, dissociates into D${}_{{{{{{{{\rm{I}}}}}}}}}^{+}$ + OD${}_{{{{{{{{\rm{II}}}}}}}}}^{+}$(a ^1^Δ). Later, the a ^1^Δ state predissociates into D${}_{{{{{{{{\rm{II}}}}}}}}}^{+}$ + O(^3^P) due to its spin-orbit coupling with the A ^3^Π state of OD${}_{{{{{{{{\rm{II}}}}}}}}}^{+}$. We are not aware of a reported lifetime for the a ^1^Δ state, however we expect it to be similar to that of the b ^1^Σ^+^ state given that both are coupled to the A ^3^Π state, and their spin-orbit matrix elements have similar magnitudes according to calculations by de Vivie et al.^25^. In summary, we have discovered another sequential fragmentation path, namely D_2_O^2+^(1 ^1^B_1_) → D${}_{{{{{{{{\rm{I}}}}}}}}}^{+}$ + OD${}_{{{{{{{{\rm{II}}}}}}}}}^{+}$(a ^1^Δ) followed by OD${}_{{{{{{{{\rm{II}}}}}}}}}^{+}$(a ^1^Δ) → D${}_{{{{{{{{\rm{II}}}}}}}}}^{+}$ + O(^3^P).

### State-selective separation of fragmentation pathways

In Fig. 5 we also compare the measured and simulated KER-correlation maps to each other. In this simulation, we assumed that the cross sections for double ionization are the same for the 2 ^1^A_1_ and 1 ^1^B_1_ states. Both features in the figure match very well, suggesting that our classical trajectory approach, though it approximates the nonadiabatic dynamics, captures the essence of the physical process. Moreover, the measured KER-correlation map enables separation between the two sequential-fragmentation pathways, thus allowing their direct comparison without recourse to theory. This separation is accomplished by selecting events to the right (2 ^1^A_1_ →  b ^1^Σ^+^) or left (1 ^1^B_1_ → a ^1^Δ) of the black-dashed line shown in Fig. 5a. Note that this line is tilted reflecting a constant KER (specifically, we used 7.18 eV in the analysis).

Our classical trajectory calculations propagate the same number of trajectories on each dication surface, with no regard to the cross section for producing that dication state in double photoionization. As a result, those calculations can accurately reflect the branching ratios between two- and three-body breakup channels on each electronic state, but provide no information about the photoionization cross sections. In contrast, the experiment does determine the ratio between the two, thus providing a test of the relative magnitudes of the cross sections to be determined by future double photoionization calculations.

### Step-by-step comparison of measured and simulated KER

**Fig. 6:**
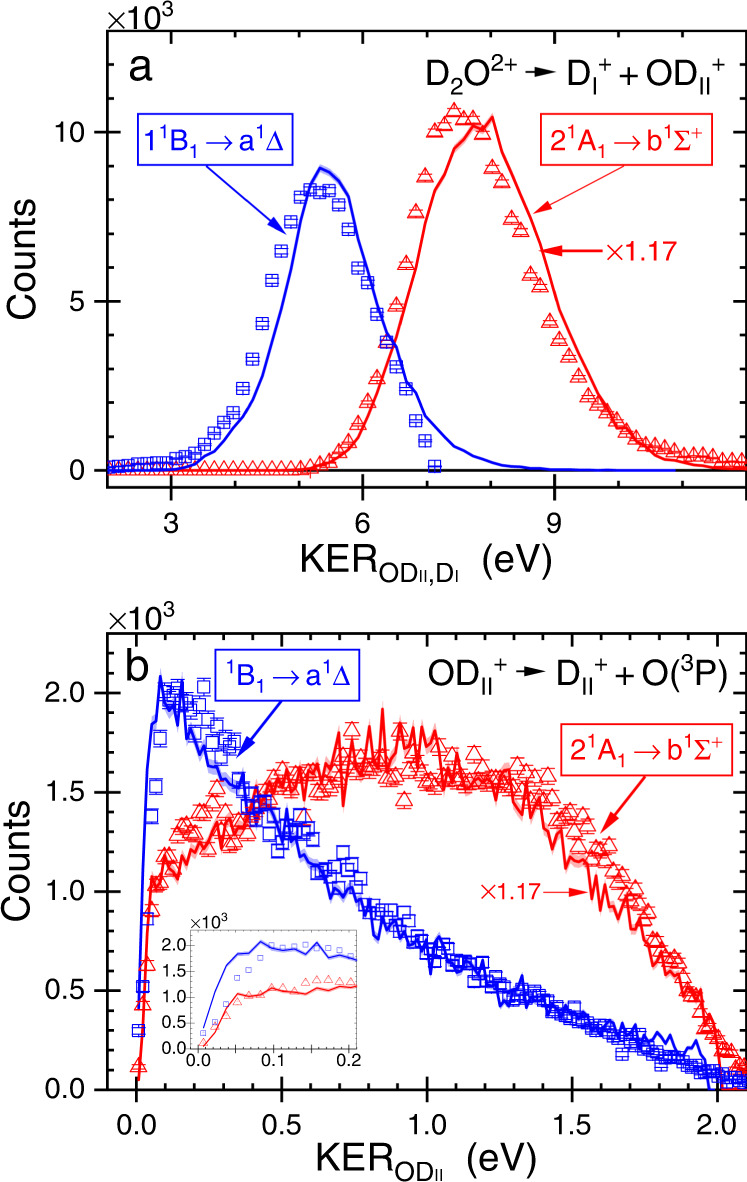
The KER distribution in each breakup step of water dications. The yield of sequential fragmentation of D_2_O^2+^ into D^+^ + D^+^ + O(^3^P) as a function of **a** KER${}_{{{{{{{{{\rm{OD}}}}}}}}}_{{{{{{{{\rm{II}}}}}}}}},{{{{{{{{\rm{D}}}}}}}}}_{{{{{{{{\rm{I}}}}}}}}}}$ and **b** KER${}_{{{{{{{{{\rm{OD}}}}}}}}}_{{{{{{{{\rm{II}}}}}}}}}}$ (Inset: Zoom-in of low energy range). Comparison of experiment (symbols and statistical error bars) and normalized theory (solid lines, with shaded range marking the simulation's statistical errors) for the two fragmentation pathways (and associated states) identified and separated using Fig. 5a (see text and Supplementary Note 1). As indicated on the figure, the theory scaling of one path is a factor of 1.17 larger than the other one (see text). The high energy cutoff in the measured 1 ^1^B_1_ to a ^1^Δ data in the upper panel is likely due to the imperfect separation of the two channels by the black-dashed line in FIg. 5. Source data are provided as a Source Data file.

In Fig. 6 we compare the measured and calculated KER distributions for both steps of each sequential fragmentation path. Because the experiment measures the relative cross sections, we scale the theory with an overall factor determined by a least squares fit to preserve the measured information on the relative likelihood of the two sequential fragmentation paths. Note that the calculated KER distribution associated with the 2 ^1^A_1_ → b ^1^Σ^+^ path is scaled by a factor that is 1.17 times larger than the scaling factor used to normalize the 1 ^1^B_1_ → a ^1^Δ path, as indicated in Fig. 6. This difference is mainly due to the relative magnitudes of the cross sections for double photoionization by a single 61 eV photon landing on the 2 ^1^A_1_ and 1 ^1^B_1_ states of D_2_O^2+^.

At a glance, the calculated and measured KER distributions associated with the D_2_O^2+^ → D${}_{{{{{{{{\rm{I}}}}}}}}}^{+}$ +OD${}_{{{{{{{{\rm{II}}}}}}}}}^{+}$ breakup, shown in Fig. 6a, match each other nicely. However, a careful inspection shows that the calculated distributions are shifted up by about 0.15 eV. We suspect that this difference stems from approximating the initial wave function of D_2_O, which is projected to the D_2_O^2+^ states, by a product of normal mode harmonic oscillators instead of a more accurate representation including the effects of the anharmonicity in the ground state PES. Similar discrepancies have been observed in H_2_ and were corrected by using a Morse potential to represent the initial state^28^.

The calculated and measured KER distribution associated with the second fragmentation step, namely OD${}_{{{{{{{{\rm{II}}}}}}}}}^{+}$ → D${}_{{{{{{{{\rm{II}}}}}}}}}^{+}$ + O shown in Fig. 6b, match very well for both sequential fragmentation paths. The differences between the KER distributions associated with these two fragmentation paths, noticeable in Fig. 5, become more apparent. The sequential fragmentation via the b ^1^Σ^+^ state yields a broad, relatively flat, KER distribution peaked around 0.9 eV, while the fragmentation via the a ^1^Δ state peaks at much lower KER, ∼0.15 eV, and falls off rapidly with increasing KER. The fact that the classical trajectory analysis faithfully reproduces the details of these distributions is a strong verification of its validity and the accuracy of the potential surfaces on which it is based.

### Internal energy of the intermediate OD${}_{{{{{{{{\rm{II}}}}}}}}}^{+}$ fragment

It is important to note that the KER in the predissociation of the OD${}_{{{{{{{{\rm{II}}}}}}}}}^{+}$ images the internal energy of this diatomic ion above the O(^3^P) dissociation limit, i.e., the internal energy stored in highly excited rovibrational states. This assertion is correct if the assumption, used in our modeling, that all rovibrational states dissociate when their energy exeeds the effective potential barrier leading to the D${}_{{{{{{{{\rm{II}}}}}}}}}^{+}$ + O(^3^P) dissociation limit, is valid. The comparison of experiment and theory in Fig. 6b validates that assumption. The remaining population, in the lower rovibrational states of the a ^1^Δ and b ^1^Σ^+^ states of OD${}_{{{{{{{{\rm{II}}}}}}}}}^{+}$, is measured as the dominant two-body breakup, D${}_{{{{{{{{\rm{I}}}}}}}}}^{+}$ + OD${}_{{{{{{{{\rm{II}}}}}}}}}^{+}$ (see previous reports on H_2_O)^23,24^.

### Angular momentum of the intermediate OD${}_{{{{{{{{\rm{II}}}}}}}}}^{+}$ molecule

In addition to the internal energy of the OD${}_{{{{{{{{\rm{II}}}}}}}}}^{+}$ ion, our classical trajectory simulations provide the angular momentum distribution, *P*(*J*), of each state of OD${}_{{{{{{{{\rm{II}}}}}}}}}^{+}$ shown in Fig. 3. Similar to the KER distributions, here too the *P*(*J*) distributions of the a ^1^Δ and b ^1^Σ^+^ states are different. The former peaks at a lower *J* (∼26) and has a long tail extending all the way to *J* = 0, while the latter peaks around *J* = 30 and is much narrower.

There are two observable consequences of these predicted *J* distributions. First, the high rotational angular momentum implies that the orientation of the long-lived OD${}_{{{{{{{{\rm{II}}}}}}}}}^{+}$ fragment will not be correlated with the direction of emission of the first D${}_{{{{{{{{\rm{I}}}}}}}}}^{+}$ fragment. This is in agreement with the nearly uniform ${\theta }_{{{{{{{{{\rm{OD}}}}}}}}}_{{{{{{{{\rm{II}}}}}}}}},{{{{{{{{\rm{D}}}}}}}}}_{{{{{{{{\rm{I}}}}}}}}}}$ angular distribution seen in the experiment displayed in Fig. 2. Second, the high rotational angular momentum of the OD${}_{{{{{{{{\rm{II}}}}}}}}}^{+}$ fragment results in a barrier with respect to the dissociation limit (i.e., centrifugal barrier) indicated in Fig. 3c, d. The internal energy of the OD${}_{{{{{{{{\rm{II}}}}}}}}}^{+}$ fragment is reflected in the distribution of KER${}_{{{{{{{{{\rm{OD}}}}}}}}}_{{{{{{{{\rm{II}}}}}}}}}}$ to which all angular momenta contribute. The threshold behavior in Fig. 6b in which the KER distribution vanishes as KER${}_{{{{{{{{{\rm{OD}}}}}}}}}_{{{{{{{{\rm{II}}}}}}}}}}$ goes to zero has its origin in the fact that only a small fraction of the OD${}_{{{{{{{{\rm{II}}}}}}}}}^{+}$ ions is produced with zero rotational angular momentum, and only this contribution can yield zero KER. This threshold behavior of the KER${}_{{{{{{{{{\rm{OD}}}}}}}}}_{{{{{{{{\rm{II}}}}}}}}}}$ distribution predicted by the trajectory calculations is seen to be in close agreement with the experimental data.

## Discussion

We studied the sequential fragmentation of water following double ionization by a single photon in unprecedented detail. The events associated with this process were separated experimentally from other fragmentation processes leading to the same final three-body channel, namely D^+^ + D^+^ + O(^3^P), using the native frames method and taking advantage of the rotation of the metastable OD${}_{{{{{{{{\rm{II}}}}}}}}}^{+}$ intermediate in the fragmentation plane. We identified two sequential-fragmentation pathways involving different electronic states and followed them step by step. Specifically, the routes are:

3 $$\begin{array}{ll}&{{{{{{{{\rm{D}}}}}}}}}_{2}{{{{{{{{\rm{O}}}}}}}}}^{2+}({2\;}^{1}{{{{{{{{\rm{A}}}}}}}}}_{1})\to {{{{{{{{\rm{D}}}}}}}}}_{{{{{{{{\rm{I}}}}}}}}}^{+}\,+\,{{{{{{{{\rm{OD}}}}}}}}}_{{{{{{{{\rm{II}}}}}}}}}^{+}({{{{{{{{\rm{b}}}}}}}}\;}^{1}{{{\Sigma }}}^{+})\quad \\ &{{{{{{{\rm{followed}}}}}}}}\,{{{{{{{\rm{by}}}}}}}}\,{{{{{{{{\rm{OD}}}}}}}}}_{{{{{{{{\rm{II}}}}}}}}}^{+}({{{{{{{{\rm{b}}}}}}}}\;}^{1}{{{\Sigma }}}^{+})\to {{{{{{{{\rm{D}}}}}}}}}_{{{{{{{{\rm{II}}}}}}}}}^{+}\,+\,{{{{{{{\rm{O}}}}}}}}{\left(\right.}^{3}\left.{{{{{{{\rm{P}}}}}}}}\right),\end{array}$$

and

4 $$\begin{array}{ll}&{{{{{{{{\rm{D}}}}}}}}}_{2}{{{{{{{{\rm{O}}}}}}}}}^{2+}({1\;}^{1}{{{{{{{{\rm{B}}}}}}}}}_{1})\to {{{{{{{{\rm{D}}}}}}}}}_{{{{{{{{\rm{I}}}}}}}}}^{+}\,+\,{{{{{{{{\rm{OD}}}}}}}}}_{{{{{{{{\rm{II}}}}}}}}}^{+}({{{{{{{{\rm{a}}}}}}}}\;}^{1}{{\Delta }})\quad \\ &{{{{{{{\rm{followed}}}}}}}}\,{{{{{{{\rm{by}}}}}}}}\,{{{{{{{{\rm{OD}}}}}}}}}_{{{{{{{{\rm{II}}}}}}}}}^{+}({{{{{{{{\rm{a}}}}}}}}\;}^{1}{{\Delta }})\to {{{{{{{{\rm{D}}}}}}}}}_{{{{{{{{\rm{II}}}}}}}}}^{+}\,+\,{{{{{{{\rm{O}}}}}}}}{\left(\right.}^{3}\left.{{{{{{{\rm{P}}}}}}}}\right).\end{array}$$

In both pathways, the second fragmentation step involves predissociation of the OD${}_{{{{{{{{\rm{II}}}}}}}}}^{+}$ due to spin-orbit coupling of the populated b ^1^Σ^+^ and a ^1^Δ states with the A ^3^Π state.

The results of our classical trajectory propagation on the D_2_O^2+^ potential surfaces are overall in excellent agreement with the experimental data associated with both sequential fragmentation paths and the steps each one undergoes, which are detailed in Eqs. (3) and (4). We draw particular attention to our ability to calculate the internal energy of the intermediate OD${}_{{{{{{{{\rm{II}}}}}}}}}^{+}$ molecule and probe it experimentally above the D${}_{{{{{{{{\rm{II}}}}}}}}}^{+}$ + O(^3^P) dissociation limit. Likewise, the angular momentum distribution of the intermediate OD${}_{{{{{{{{\rm{II}}}}}}}}}^{+}$ molecule has been computed and the predicted impact on the low KER distribution has been measured.

Though our methodology has been demonstrated for sequential fragmentation of water following double ionization by a single photon, it is not limited to this specific case, neither is it the only available methodology as covariance imaging analysis may also provide accurate results^29^. In our methodology, the combination of kinematically complete momentum imaging measurements, classical trajectory simulations on the relevant PES, and the native frames method should be applicable to a wide range of polyatomic molecules as long as the energy deposited in the system is known and there is a clear signature enabling identification of sequential fragmentation from other processes, like the rotation of the intermediate molecular fragment in this case. This methodology enhances our capabilities for exploring molecular reaction dynamics on the PES around asymmetric stretch that is commonly the path for forming an intermediate molecular fragment en route to sequential fragmentation. The observation of sequential steps in a molecular dissociation reaction via their unambiguous signature in the momenta of the fragments has added the dimension of time evolution to an essentially time-independent measurement.

## Methods

The methodology that enables us to compare theory and experiment step by step and state selectively is based on three main components, described briefly below. Further details are provided in the references.

### Theory

The PESs of the water dication are computed using the MOLPRO^30,31^ quantum chemistry suite in internally contracted multireference configuration interaction (icMRCI) methods at the configuration interaction singles and doubles level relative to the reference space including Davidson correction to the energy, by Gervais et al.^26^ and Streeter et al.^23^. The full dimensional surfaces are then fitted using a linear least squares fit to a functional form developed by Gervais et al.^26^. Those studies established the branching ratios between two-body (D^+^ + OD^+^) and three-body (D^+^ + D^+^ + O) breakup on each potential surface of the nine states of the water dication that can be accessed at the photon energy used in our experiment.

Three states of D_2_O^2+^, the 1 ^1^A_1_, 2 ^1^A_1_, and 1 ^1^B_1_ states, shown in Fig. 1a, produce the b ^1^Σ^+^ state (which correlates with the 2 ^1^A_1_ state) and the two components of the degenerate a ^1^Δ state of OD${}_{{{{{{{{\rm{II}}}}}}}}}^{+}$^25,26^, both shown in Fig. 1b. However, only two of those, the 2 ^1^A_1_ and 1 ^1^B_1_ states, produce the diatomic ion with enough internal energy to dissociate appreciably by this mechanism and be seen in one-photon double photoionization, as is suggested by Fig. 5 of ref. 26.

We model the reaction dynamics by propagating ensembles of classical trajectories on the relevant potential surface for a few picoseconds and then evaluate the OD${}_{{{{{{{{\rm{II}}}}}}}}}^{+}$ population. The computational approach used here is the same as that of our previous studies^23,24^. The Wigner phase space density for the initial vibrational state is propagated on the dication PESs using classical trajectories, and the final momenta and internal energy distributions are evaluated classically from this ensemble of trajectories. This approach has been benchmarked for three-body breakup in this system by excellent agreement of the final momentum distribution with momentum imaging experiments^23,24^. For the internal energy distributions of the OH^+^ fragment in the two-body channels, the accuracy of the classical Wigner method was confirmed by Gervais et al.^26^ as well as by the agreement with the experiment displayed in Fig. 6. Such an ensemble of trajectories is the basis of the well-studied Wigner semiclassical method^32–34^ for predicting final quantum state distributions. For the dissociative dynamics in this case, however, quantum effects are unimportant and a purely classical analysis suffices.

The molecule is initially in its ground vibrational state and undergoes a Franck–Condon transition to the doubly ionized excited state. The initial conditions for the 1,000,000 computed classical trajectories, for each D_2_O^2+^ state we consider, are sampled from the corresponding Wigner distribution, assuming that the ground vibrational state can be represented by a direct product wave function of the normal modes. At the end of the propagation the internal energy, and the rotational angular momentum, *J*, of the OD${}_{{{{{{{{\rm{II}}}}}}}}}^{+}$ fragment, are computed from the Cartesian momenta and coordinates of the atoms together with the value of the potential energy. The OD${}_{{{{{{{{\rm{II}}}}}}}}}^{+}$ is considered to be bound if the total energy of an O–D${}_{{{{{{{{\rm{II}}}}}}}}}^{+}$ pair is less than the *J*-dependent barrier to dissociation associated with the relevant final states of OD${}_{{{{{{{{\rm{II}}}}}}}}}^{+}$, specifically the D${}_{{{{{{{{\rm{II}}}}}}}}}^{+}$ + O(^1^D) limit shown in Fig. 1b.

As stated above, the second fragmentation step involves predissociation of the b ^1^Σ^+^ and a ^1^Δ states of the intermediate OD${}_{{{{{{{{\rm{II}}}}}}}}}^{+}$ ion due to spin-orbit coupling with its A ^3^Π state. The central dynamical assumption of our treatment takes advantage of the relatively short lifetimes for this predissociation via a nonadiabatic transition to the A ^3^Π state compared to the fragments’ flight time in the experimental setup. We therefore assume in our classical trajectory calculations that all OD${}_{{{{{{{{\rm{II}}}}}}}}}^{+}$ ions, with internal energy above the D${}_{{{{{{{{\rm{II}}}}}}}}}^{+}$ + O(^3^P) dissociation limit, predissociate within a few picoseconds^25,26^. For example, all trajectories in the b ^1^Σ^+^ state having zero rotational angular momentum and with vibrational energies between the two horizontal dashed lines in Fig. 1b, labeled appearance window, are assumed to lead to D${}_{{{{{{{{\rm{II}}}}}}}}}^{+}$ + O fragments. Such an appearance window was recently found to play an important role in another polyatomic molecule, i.e., in the valence photo-double ionization of ammonia^35^. Nonzero rotational angular momentum adds a centrifugal barrier to this picture for all three states of OD${}_{{{{{{{{\rm{II}}}}}}}}}^{+}$ in Fig. 1b, modifying the assumption about the dissociating rovibrational states to those with energy above the barrier leading to the D${}_{{{{{{{{\rm{II}}}}}}}}}^{+}$ + O(^3^P) dissociation limit, and therefore the appearance window shifts. For simplicity, tunneling through the centrifugal barrier for *J* ≠ 0 is neglected in our treatment.

### Experimental method

To identify, separate, and follow all electronic states involved in the dissociation sequence, a kinematically complete experiment is required, and that entails the determination of the final momenta of all fragments from each molecule. In the present case, we measure two electrons and two deuterons in coincidence and determine their final momenta by solving their classical equations of motion in the COLTRIMS setup^5–7^. Then, we employ momentum conservation to evaluate the final momentum of the neutral oxygen. To avoid artifacts caused by the identical D^+^ fragments, we follow the common practice and randomize their time order, i.e., flip the order of the 1st and 2nd D^+^ hits randomly for half of the events. It is worth noting that the few picoseconds lifetime of the OD^+^ intermediate molecule is long enough to completely deplete the populated OD^+^ states above the D^+^ + O(^3^P) dissociation limit, and short enough (sub nanosecond) to avoid distortion of the measured momentum images.

It is also essential to know how much energy is deposited in each water molecule. To accomplish that we doubly ionize the water molecule by single-photon absorption, using a 61 eV narrow bandwidth (50 meV) synchrotron light pulse provided by the Advanced Light Source (ALS), thereby producing water dications 24.1 eV above the D^+^ + D^+^ + O(^3^P) dissociation limit. Using this photon energy together with the measured kinetic energy of both electrons and all the other fragments allows the selection of only events ending on a specific dissociation limit^23,24^. Using 61 eV photons, which are below the triple ionization potential of water, also eliminates the need to filter out D^+^ + D^+^ + O^+^ events.

The expected total energy release, *E*_release_, is computed by subtracting the complete dissociation energy of the heavy water dication into D^+^ + D^+^ + O(^3^P) from the measured photon energy used in our experiment. The accumulated error in the measured *E*_release_ and photon energy, as well as uncertainties in the complete dissociation energy of water recommended by NIST, add up to an estimated uncertainty of the order of the energy shift needed to match the measured spectrum shown in Fig. 4 (see Supplementary Note 2 for further details).

Heavy water, D_2_O, is chosen over H_2_O in order to circumvent contributions from double ionization of H_2_O and H_2_ molecules, both common residual gases in ultra-high vacuum systems, also producing H^+^ + H^+^+ 2*e*^−^. These residual gases are much warmer than the COLTRIMS jet and are present throughout the light-beam propagation direction, therefore contaminating the momentum imaging, especially when one cannot use momentum conservation to eliminate them, as is the case for the breakup channel of interest. The D_2_O target choice should not affect the fragmentation pathways since each water isotopologue is expected to undergo similar dynamics.

### Native frames analysis

In the native frames method^21,22^, which we employ to analyze the three-body breakup, the key ingredient is the use of the conjugate momenta of the Jacobi coordinates, where these coordinates describe the relative positions of the three fragments. In the case of D_2_O, shown schematically in Fig. 2b, the conjugate momentum corresponding to the first fragmentation step is given by

5 $${{{{{{{{\bf{p}}}}}}}}}_{{{{{{{{{\rm{OD}}}}}}}}}_{{{{{{{{\rm{II}}}}}}}}},{{{{{{{{\rm{D}}}}}}}}}_{{{{{{{{\rm{I}}}}}}}}}}=\frac{{m}_{{{{{{{{\rm{OD}}}}}}}}}}{M}{{{{{{{{\bf{P}}}}}}}}}_{{{{{{{{{\rm{D}}}}}}}}}_{{{{{{{{\rm{I}}}}}}}}}}-\frac{{m}_{{{{{{{{\rm{D}}}}}}}}}}{M}\left[{{{{{{{{\bf{P}}}}}}}}}_{{{{{{{{{\rm{D}}}}}}}}}_{{{{{{{{\rm{II}}}}}}}}}}+{{{{{{{{\bf{P}}}}}}}}}_{{{{{{{{\rm{O}}}}}}}}}\right]\,,$$

where **P**_D_ and **P**_O_ are the measured momenta of the D^+^ and O fragments, respectively, *m*_D_ is the mass of D^+^, *m*_OD_ is the mass of OD_II_, and *M* is the mass of the D_2_O^2+^.

Similarly, the conjugate momentum associated with the second step is

6 $${{{{{{{{\bf{p}}}}}}}}}_{{{{{{{{{\rm{OD}}}}}}}}}_{{{{{{{{\rm{II}}}}}}}}}}={\mu }_{{{{{{{{\rm{OD}}}}}}}}}\left[\,\frac{{{{{{{{{\bf{P}}}}}}}}}_{{{{{{{{{\rm{D}}}}}}}}}_{{{{{{{{\rm{II}}}}}}}}}}}{{m}_{{{{{{{{\rm{D}}}}}}}}}}-\frac{{{{{{{{{\bf{P}}}}}}}}}_{{{{{{{{\rm{O}}}}}}}}}}{{m}_{O}}\,\right],$$

where *μ*_OD_ is the reduced mass of OD_II_. Finally, the angle between the two conjugate momenta, ${\theta }_{{{{{{{{{\rm{OD}}}}}}}}}_{{{{{{{{\rm{II}}}}}}}}},{{{{{{{{\rm{D}}}}}}}}}_{{{{{{{{\rm{I}}}}}}}}}}$, is computed from the dot product of these vectors. Equations (5) and (6) are the conjugate momenta of the Jacobi coordinates for the D_I_ + OD_II_ arrangement, and they can be used to compute the KER in each fragmentation step. For example, the KER in the second step (used in Fig. 2) is given by KER${}_{{{{{{{{{\rm{OD}}}}}}}}}_{{{{{{{{\rm{II}}}}}}}}}}={{{{{{{{\bf{p}}}}}}}}}_{{{{{{{{{\rm{OD}}}}}}}}}_{{{{{{{{\rm{II}}}}}}}}}}^{2}\,/\,2\,{\mu }_{{{{{{{{\rm{OD}}}}}}}}}$.

The second ingredient needed to separate sequential fragmentation from other competing processes is a clear signature distinguishing it from the others. As mentioned above, rotation of the intermediate OD${}_{{{{{{{{\rm{II}}}}}}}}}^{+}$ in the fragmentation plane, persisting long enough to wipe out any initial angular preference, leads to a distinct nearly uniform $N({\theta }_{{{{{{{{{\rm{OD}}}}}}}}}_{{{{{{{{\rm{II}}}}}}}}},{{{{{{{{\rm{D}}}}}}}}}_{{{{{{{{\rm{I}}}}}}}}}})$ angular distribution. The KER in the second fragmentation step, KER${}_{{{{{{{{{\rm{OD}}}}}}}}}_{{{{{{{{\rm{II}}}}}}}}}}$, combined with the molecular structure of this intermediate molecule, provides an additional constraint for identifying sequential breakup. Therefore, to identify and separate the sequential fragmentation via an OD${}_{{{{{{{{\rm{II}}}}}}}}}^{+}$ intermediate, we plot in Fig. 2a all the measured D^+^ + D^+^ + O events as a function of KER${}_{{{{{{{{{\rm{OD}}}}}}}}}_{{{{{{{{\rm{II}}}}}}}}}}$ and ${\theta }_{{{{{{{{{\rm{OD}}}}}}}}}_{{{{{{{{\rm{II}}}}}}}}},{{{{{{{{\rm{D}}}}}}}}}_{{{{{{{{\rm{I}}}}}}}}}}$, where we arbitrarily designate one of the two D^+^ fragments (denoted D${}_{{{{{{{{\rm{I}}}}}}}}}^{+}$)—ejected first—a correct coin-flip assignment for half of the sequential events. Then, we identify the (properly assigned) sequential breakup as the uniform angular stripe marked by the dashed-magenta rectangular boundary in the figure. Note that the KER distribution of this stripe matches the predicted 0–2.25 eV range^25,26^, indicated by the appearance window in Fig. 1b.

Then, we use the identified sequential events within the dashed-magenta boundary in Fig. 2a to create equivalent events by randomly rotating them to smaller angles until the whole range has a uniform yield within the uncertainty of the data. It is important to note that any other information from each event, like KER${}_{{{{{{{{{\rm{OD}}}}}}}}}_{{{{{{{{\rm{II}}}}}}}}}}$, is preserved by this reconstruction algorithm^22^. The resulting complete reconstructed angular distribution of the D_2_O^2+^ → D${}_{{{{{{{{\rm{I}}}}}}}}}^{+}$ + OD${}_{{{{{{{{\rm{II}}}}}}}}}^{+}$ followed by OD${}_{{{{{{{{\rm{II}}}}}}}}}^{+}$ → D${}_{{{{{{{{\rm{II}}}}}}}}}^{+}$ + O sequential fragmentation is shown in Fig. 2c.

Finally, in Fig. 2d we present the *N*(KER${}_{{{{{{{{{\rm{OD}}}}}}}}}_{{{{{{{{\rm{I}}}}}}}}}}$,${\theta }_{{{{{{{{{\rm{OD}}}}}}}}}_{{{{{{{{\rm{I}}}}}}}}},{{{{{{{{\rm{D}}}}}}}}}_{{{{{{{{\rm{II}}}}}}}}}}$) distribution—a similar distribution to that shown in panel (c) but with the D^+^ fragmentats associated incorrectly with their breakup-step order. Specifically, we are plotting sequential breakup occurring via the OD${}_{{{{{{{{\rm{II}}}}}}}}}^{+}$ intermediate in the frame assuming an OD${}_{{{{{{{{\rm{I}}}}}}}}}^{+}$ intermediate. To generate such a distribution with certainty, we use the events identified as sequential fragmentation via D${}_{{{{{{{{\rm{I}}}}}}}}}^{+}$ + OD${}_{{{{{{{{\rm{II}}}}}}}}}^{+}$ and shown in Fig. 2c, but analyze them as if the D${}_{{{{{{{{\rm{II}}}}}}}}}^{+}$ was ejected first, i.e., using the reference frames that are not associated with the relevant center-of-mass of each breakup step. In other words, their momenta are calculated in the other Jacobi coordinate arrangement, namely ${{{{{{{{\bf{p}}}}}}}}}_{{{{{{{{{\rm{OD}}}}}}}}}_{{{{{{{{\rm{I}}}}}}}}},{{{{{{{{\rm{D}}}}}}}}}_{{{{{{{{\rm{II}}}}}}}}}}$ and ${{{{{{{{\bf{p}}}}}}}}}_{{{{{{{{{\rm{OD}}}}}}}}}_{{{{{{{{\rm{I}}}}}}}}}}$. One can clearly see that the two distributions, shown separately in panels (c) and (d) are significantly different from each other, and both are also visible in the all-events data shown in panel (a). Hence, one can associate each detected D^+^ with the relevant fragmentation step.

## Supplementary information

Supplementary Information

## Data Availability

The data that support the findings of this study are available in this article and its Supplementary Information. The raw data are available from the corresponding authors upon request. Source data are provided with this paper.

## Source data

Source Data
