# Supplementary material for: Step-by-step state-selective tracking of fragmentation dynamics of water dications by momentum imaging
Source: Nat Commun. 2022 Sep 1;13:5146. doi: 10.1038/s41467-022-32836-6 (PMC9437093; doi:10.1038/s41467-022-32836-6)
Supplement: Supplementary file 1 — Supplementary Information [file 41467_2022_32836_MOESM1_ESM.pdf]

Supplementary Information

Step-by-step state-selective tracking of fragmentation dynamics of water dications by momentum imaging

Travis Severt, Zachary L. Streeter, Wael Iskandar, Kirk A. Larsen, Averell Gatton, Daniel Trabert, Bethany Jochim, B. Griffin, E. G. Champenois, M. M. Brister, D. Reedy, D. Call, R. Strom, Allen L. Landers, Reinhard Dörner, Joshua B. Williams, Daniel S. Slaughter, Robert R. Lucchese, Thorsten Weber, C. William McCurdy, and Itzik Ben-Itzhak

Supplementary Note 1. Pathway separation

As stated in the paper, our pathway separation method is simplified in practice by rotating Fig. 5 (from the paper) by 45°, that is we plot in Fig. (a) the same data as a function of the KER difference and KER sum, namely

$$\mathrm{KER_{diff}} = \mathrm{KER}_{\mathrm{OD_{II},D_I}} - \mathrm{KER}_{\mathrm{OD_{II}}}$$

and

$$\mathrm{KER_{sum}} = \mathrm{KER}_{\mathrm{OD_{II},D_I}} + \mathrm{KER}_{\mathrm{OD_{II}}} ,$$

respectively.

Next, we project Fig. (a) onto the $\mathrm{KER_{sum}}$ axis, as shown in Fig. (b) (note that $\mathrm{KER_{sum}}$ is the total kinetic energy release in the fragmentation process, denoted simply as KER).

Finally, we separate events larger than KER = 7.18 eV to one fragmentation pathway, $2\,^1A_1 \rightarrow b\,^1\Sigma^+$, while events with smaller KER are associated with the other breakup pathway, $1\,^1B_1 \rightarrow a\,^1\Delta$.

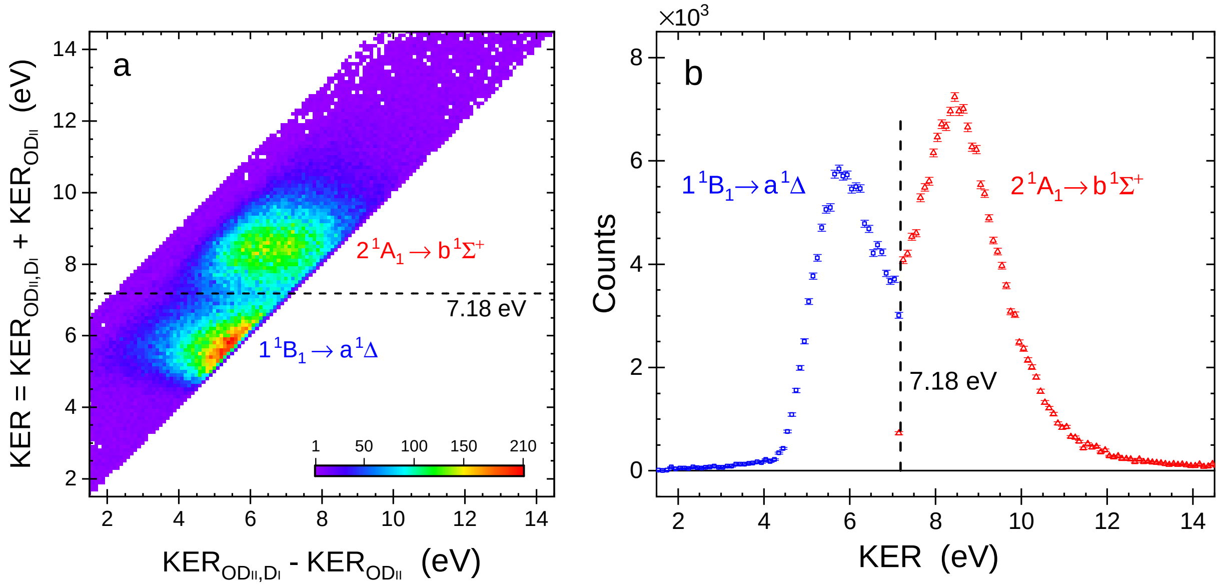

Supplementary Fig. 1 Pathway separation method. (a) Figure 5 in the paper is rotated by 45°, thus exhibiting the sequential fragmentation events as a function of KER difference [$\mathrm{KER}_{\mathrm{OD_{II},D_I}} - \mathrm{KER}_{\mathrm{OD_{II}}}$] and KER sum [$\mathrm{KER}_{\mathrm{OD_{II},D_I}} + \mathrm{KER}_{\mathrm{OD_{II}}}$]. (b) a projection of panel (a) onto the KER sum axis, i.e., showing the counts (with statistical error bars) as a function of the total KER. The vertical line at 7.18 eV separates between the two fragmentation pathways (see text), specifically the pathways are: $2\,^1A_1 \rightarrow b\,^1\Sigma^+$ (red) and $1\,^1B_1 \rightarrow a\,^1\Delta$ (blue). Note that the overlap between the two distributions around the vertical line is small. Source data are provided as a Source Data file.

Supplementary Note 2. Energy release

In Fig. 4 of the paper we compare the measured energy release, $E_{\rm release}$, following double ionization of heavy water by a 61-eV photon and leading to $D^{+}+D^{+}+O$ three-body fragmentation, with the expected values. Recall that the measured $E_{\rm release}$ is given by

$$E_{\rm release} = {\rm KER} + E_{e_1} + E_{e_2}\,, \qquad (1)$$

where KER is the total kinetic energy carried by the three massive fragments, and $E_{e_1} + E_{e_2}$ is the kinetic energy of both electrons in the continuum. The measured spacing between the different dissociation limits associated with the oxygen ^{3}P ground state and the ^{1}D and ^{1}S excited states, match well the expected values, while matching the absolute values is more challenging. To circumvent such mismatch, it became common practice to show only the relative $E_{\rm release}$ values, that is, the energy spacing when comparing measured spectrum and expected values [1].

To match our measured $E_{\rm release}$ spectrum, shown in Fig. 4 of the paper, we have to downshift by 0.4 eV the expected value for the lowest dissociation limit, i.e., the $D^{+}+D^{+}+O(^3P)$ limit, denoted hereafter by the expression $E[D^{+} + D^{+} + O(^3P)]$. It is important to note that this expected value is based on recommended measured values as explained below. The expected value of the energy release, denoted $E'_{\rm release}$ to distinguish it from the measured value, is given by

$$E'_{\rm release} = E_{\rm photon} - E[{\rm D}^{+} + {\rm D}^{+} + {\rm O}(^3{\rm P})]\,, \qquad (2)$$

where $E_{\rm photon}$ is the photon energy dialed in at the monochromator of the synchrotron beamline. Note that in this context we need to know the energy of the lowest dissociation limit, $E[D^{+} + D^{+} + O(^3P)]$, relative to the $D_2O(v_1, v_2, v_3 = 0{,}0{,}0)$ ground state energy.

This dissociation energy, namely $E[D^{+}+D^{+}+O(^3P)]$, is evaluated by adding the NIST [2] recommended values for the dissociation of H–OH, 5.098 eV [3], and O–H, 4.414 eV [3] to twice the ionization potential of the hydrogen atom, 13.59844 eV [2], Then, correcting for the difference between the zero-point energy of the D_2O and H_2O, which is 0.15461 eV [specifically, using the values from Ref. [4]:

$(4638.6\,{\rm cm}^{-1}$ - $3391.6\,{\rm cm}^{-1}) \times 1.2398{\times}10^{-4}\,{\rm eV/cm}^{-1}$
$= 0.15461\,{\rm eV}$,

yields

$$E[{\rm D}^{+} + {\rm D}^{+} + {\rm O}(^3{\rm P})] = 36.8638\,{\rm eV} \qquad (3)$$

above the $D_2O(v_1, v_2, v_3 = 0{,}0{,}0)$ ground state. Finally, given the photon energy of 61.0 eV, the expected energy release associated with the $D^{+}+D^{+}+O(^3P)$ dissociation limit should be

$$E'_{\rm release} = 61.0 - 36.8638 = 24.1362\,{\rm eV}. \qquad (4)$$

As stated in the paper, the measured energy release, $E_{\rm release}$, associated with this dissociation limit is about 0.4 eV lower than the expected value, $E'_{\rm release}$. Though an absolute determination of this energy was not the goal in our experiment one may wonder what is the source for this mismatch. The scatter in the recommended values across measurements is of the order of 100 meV, i.e., much smaller than the shift. The uncertainties in our measurements originate from the calibration of the measured photon energy via indirect measurements [mostly angle-resolved photoemission spectroscopy (ARPES)] — estimated to be 50-150 meV, the energy calibration of the electrons of about 50 meV (at around 20 eV), while the two ions contribute about 200 meV due to calibration using previous N_2 data [5]. Therefore, the absolute energy shift used in Fig. 4 is within the combined uncertainty of our measurement and the scatter in the recommended data.

In principle, though with significant effort, the uncertainties in our measurements can be reduced to enable accurate determination of the absolute energy. However, to identify the final states of the fragmentation process it is sufficient to use the relative energies (i.e., energy spacings between the peaks), and one would expect them to always be more precise, as they are independent of some of the systematic errors, like the accuracy of the beamline monochromator calibration or uncertainties in the dissociation energies.

Supplementary References

1. D. Reedy, J. B. Williams, B. Gaire, A. Gatton, M. Weller, A. Menssen, T. Bauer, K. Henrichs, P. Burzynski, B. Berry, Z. L. Streeter, J. Sartor, I. Ben-Itzhak, T. Jahnke, R. Dörner, T. Weber, and A. L. Landers, Dissociation dynamics of the water dication following one photon double ionization. II. Experiment, Phys. Rev. A **98**, 053430 (2018).
2. https://www.nist.gov/pml/productsservices/physical-reference-data.
3. B. Ruscic, D. Feller, D. A. Dixon, K. A. Peterson, L. B. Harding, R. L. Asher, and A. F. Wagner, Evidence for a lower enthalpy of formation of hydroxyl radical and a lower gas-phase bond dissociation energy of water, J. Phys. Chem. A **105**, 1 (2001).
4. J. R. Hulston, Revised zero-point energy calculation for $H_2O + D_2O \rightleftarrows$ HDO isotopic equilibrium, J. Chem. Phys. **50**, 1483 (1969).
5. M. Lundqvist, D. Edvardsson, P. Baltzer, and B. Wannberg, Doppler-free kinetic energy release spectrum of N_2^{2+}, J. Phys. B **29**, 1489 (1996).
